# Seats of Tubercle

**Published:** 1844-01

**Authors:** 


*Seats of Tubercle.*—Laennec believed that the right lung was the most frequent seat of tubercles; Louis thought otherwise. Of 250 cases of pulmonary tubercle recorded by Dr. Hughes (Guy's Hospital Reports), in 166 the left lung was found chiefly diseased, and the right in 89, while in 45 cases doubt existed as to which was the most diseased side. It would appear from the above partial experience that, contrary to the opinion of Laennec, the left lung is the most liable to tuberculary deposit. Of the 116 cases of disease in the left lung, 43 per cent. occurred in males, and 53 per cent. in females; while of the 89 cases of disease in the right side, 38 per cent. were in males, and 30 per cent. in females; therefore females would appear to be most liable to tubercle in the left, and males to tubercle in the right lung. Of the total 250 cases, the upper lobe of one or both lungs was solely or principally diseased in 237, or 95 per cent.; of the remaing 13, in 9 cases both lungs were uniformly diseased; and in only one case were the upper lobes free from tubercles. Of the same total number the per-centage of the sexes was about equal in deaths from tubercular phthisis under twenty years of age (viz: 8 per cent. of males, and 7 per cent. females); of those dying between tne ages of twenty and thirty, 41 per cent, were males, and 49 per cent. females; of those between thirty and forty, the per-centage was again nearly equal (viz: 26 per cent. for males, and 27 per cent. for females); and between forty and fity, the deaths of males were 23 per cent., and those of females 19. It would thus result that females were comparitively more liable to die of phthisis between the ages of twenty and thirty, and males between forty and fifty. *London Lancet.*

